# Pro-fibrinolytic potential of the third larval stage of *Ascaris suum* as a possible mechanism facilitating its migration through the host tissues

**DOI:** 10.1186/s13071-020-04067-5

**Published:** 2020-04-20

**Authors:** Alicia Diosdado, Fernando Simón, Rodrigo Morchón, Javier González-Miguel

**Affiliations:** 1 Laboratory of Parasitology, Faculty of Pharmacy, University of Salamanca, C/Licenciado Méndez Nieto s/n, 37007 Salamanca, Spain; 2 Laboratory of Parasitology, Institute of Natural Resources and Agrobiology of Salamanca (IRNASA-CSIC), C/Cordel de Merinas 40-52, 37008 Salamanca, Spain; 3 Martsinovsky Institute of Medical Parasitology, Tropical and Vector Borne Diseases, Sechenov University, Malaya Pirogovskaya St. 20-1, Moscow, 119435 Russia

**Keywords:** *Ascaris suum*, Third-stage larvae, Fibrinolytic system, Plasminogen, Plasmin, Larval migration, Ascariasis, Host-parasite relationships

## Abstract

**Background:**

*Ascaris* roundworms are the parasitic nematodes responsible for causing human and porcine ascariasis. Whereas *A. lumbricoides* is the most common soil-transmitted helminth infecting humans in the world, *A. suum* causes important economic losses in the porcine industry. The latter has been proposed as a model for the study of *A. lumbricoides* since both species are closely related. The third larval stage of these parasites carries out an intriguing and complex hepatopulmonary route through the bloodstream of its hosts. This allows the interaction between larvae and the physiological mechanisms of the hosts circulatory system, such as the fibrinolytic system. Parasite migration has been widely linked to the activation of this system by pathogens that are able to bind plasminogen and enhance plasmin generation. Therefore, the aim of this study was to examine the interaction between the infective third larval stage of *A. suum* and the host fibrinolytic system as a model of the host-*Ascaris* spp. relationships.

**Methods:**

Infective larvae were obtained after incubating and hatching fertile eggs of *A. suum* in order to extract their cuticle and excretory/secretory antigens. The ability of both extracts to bind and activate plasminogen, as well as promote plasmin generation were assayed by ELISA and western blot. The location of plasminogen binding on the larval surface was revealed by immunofluorescence. The plasminogen-binding proteins from both antigenic extracts were revealed by two-dimensional electrophoresis and plasminogen-ligand blotting, and identified by mass spectrometry.

**Results:**

Cuticle and excretory/secretory antigens from infective larvae of *A. suum* were able to bind plasminogen and promote plasmin generation in the presence of plasminogen activators. Plasminogen binding was located on the larval surface. Twelve plasminogen-binding proteins were identified in both antigenic extracts.

**Conclusions:**

To the best of our knowledge, the present results showed for the first time, the pro-fibrinolytic potential of infective larvae of *Ascaris* spp., which suggests a novel parasite survival mechanism by facilitating the migration through host tissues.
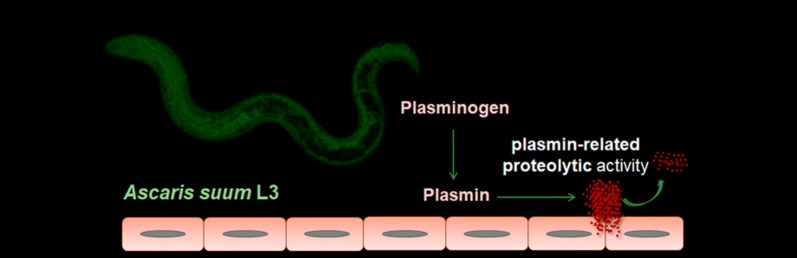

## Background

*Ascaris suum*, generally considered as the most prevalent intestinal parasite in domestic pigs, is the nematode responsible for causing porcine ascariasis. This soil-transmitted infection presents high prevalence rates around the world and entails significant economic losses in the swine industry due to the reduction of production efficiency and organ condemnations [1]. In addition, *A. suum* has been postulated as a model for the study of *A. lumbricoides* infection in humans, which involves a parasitosis affecting an estimated 804 million people, most commonly children and adolescents [2, 3]. One of the most striking characteristics of the life-cycle of these parasites is the complex migratory route carried out by their third-stage larvae (L3) before establishing in the intestine. After being released from the eggs in the small intestine, larvae head for the caecum and proximal colon to undertake a hepatopulmonary migration through the bloodstream. Once L3 reach the alveoli, they ascend the trachea in order to be swallowed through the oesophagus and return to the small intestine, where they eventually reach the adult stage [4]. Despite being a process with high adaptive cost for the parasite, it could confer evolutionary benefits in terms of establishment in the host, as it has been proposed for nematode parasites whose larvae undergo migrations that begin and end in the same location [5]. However, the molecular mechanisms governing L3 migration processes in ascariasis remain unclear [6, 7]. For this reason, the knowledge of these key aspects of the life-cycle of the parasite could contribute to develop new intervention strategies in human and porcine ascariasis by studying the molecular basis of the host-parasite relationships [7, 8]. Thereby, the interaction between different species of pathogens and the fibrinolytic system of their corresponding host has already been studied [9, 10].

The fibrinolytic system is the main mechanism responsible for degrading blood clots in mammals [11]. Its activity is based on the conversion of a circulating zymogen in plasma (plasminogen) into its proteolytically active enzyme (plasmin). Plasminogen possesses lysine-binding sites called kringle domains, which interact with the lysine residues of different proteins and cellular receptors. Plasminogen is transformed into plasmin by cleavage of a peptide bond by the action of two proteases, the tissue plasminogen activator (tPA) and the urokinase-type plasminogen activator (uPA) [12]. The serine protease plasmin generated exerts its proteolytic activity against a broad range of substrates, including fibrin of blood clots and different components of the extracellular matrix [13]. Consequently, plasminogen recruitment and activation to plasmin by secreted and surface proteins of many groups of bacteria, fungi and parasites have been described and related to their invasion and migration processes, among others, facilitating their establishment in the host [9, 10, 14, 15].

In order to contribute to the knowledge of the host-*Ascaris* spp. relationships, especially during the parasitic intraorganic migration, the aim of this study was to examine the interaction between the cuticle and excretory/secretory antigenic extracts of the L3 of *A. suum* (AsL3C and AsL3ES) and the host fibrinolytic system.

## Methods

### Collection of third-stage larvae of *A. suum*

AsL3 were obtained following the protocol described by Vlaminck et al. [16] with minor modifications. Adult female worms of *A. suum* were collected from the intestines of naturally infected pigs from a local abattoir and dissected in order to extract the uterus and obtain the eggs. All the parasite material was washed with phosphate-buffered saline (PBS), pH 7.2. The eggs were suspended in a 2% potassium dichromate (K_2_Cr_2_O_7_) solution and placed in culture plates for their incubation at 27 °C for approximately 40 days in a place restricted from light. The incubation process was monitored by light microscopy observation.

Once most of the eggs were embryonated, they were collected from culture plates and transferred to test tubes to be treated with 5–6% sodium hypochlorite (NaClO) (commercial bleach) at 37 °C for 15 min. The eggs were washed at least 5 times with PBS by centrifugation at 200× *g* for 5 min. The suspension was transferred into an Erlenmeyer flask containing glass beads and a magnetic stir bar and maintained for approximately 1 h shaking slowly (60× *rpm*). Egg hatching was monitored by observing the solution under an optical microscope. After most of the larvae were outside the egg, the suspension was poured into a cotton gauze layer (2.5 g) on a Baermann apparatus filled with PBS and maintained at 37 °C and 5% CO_2_ overnight in a place restricted from light following the method described by Urban et al. [17]. Larvae were then collected from the neck of the funnel and washed with PBS. Some of them were used to obtain the antigenic extracts subsequently described, while others were fixed in a solution of 10% formaldehyde until use.

### Collection of cuticle and excretory/secretory antigenic extracts from *A. suum* third-stage larvae

AsL3C and AsL3ES extracts were obtained following the methodology described by Wedrychowicz et al. [18] and González-Miguel et al. [19] with some modifications. In order to isolate the cuticle antigens from the AsL3, these larvae were incubated in a saline solution containing 0.25% cetyl trimethyl ammonium bromide (CTAB) at 37 °C for 4 h shaking. After removing larvae by centrifugation at 200× *g* for 5 min, the supernatant was filtered through a filter of 0.22 µm. Proteins were precipitated with a solution of 0.002 M sodium acetate and 9 volumes of 96% ethanol at − 20 °C for 48 h. The suspension was centrifuged at 10.000× *g* for 10 min and the resulting pellet was re-suspended in PBS. The same quantity of AsL3 was cultured in RPMI-1640 medium (Sigma-Aldrich, St. Louis, USA) supplemented with an antibiotic-antimycotic solution (100×) (Sigma-Aldrich) at 37 °C and 5% CO_2_ for 24 h to obtain the excretory/secretory products. Then, the suspension was centrifuged at 200× *g* for 5 min to remove larvae, and the resulting medium was filtered through a filter of 0.22 µm. After dialyzing against water at 4 °C for 48 h shaking, proteins were concentrated using Amicon Ultra-15 centrifugal filter devices (Millipore, Burlington, USA). Finally, both extracts were mixed with a cocktail of proteases inhibitors [20], their protein concentrations measured by BCA protein assay reagent kit (Thermo Fisher Scientific, Waltham, USA) and stored at − 80 °C until their use.

### Plasminogen binding assays

The ability of AsL3C and AsL3ES to bind plasminogen was studied by an enzyme-linked immunosorbent assay (ELISA) according to the methods by González-Miguel et al. [19] with minor modifications. In brief, multi-well microplates (Corning, New York, USA) were coated with 1 µg/well of AsL3C or AsL3ES diluted in carbonate buffer, pH 9.6, at 4 °C overnight and blocked with 1% bovine serum albumin (BSA) diluted in PBS at 37 °C for 30 min. Wells were successively incubated with increasing amounts (0–2 µg/well) of human plasminogen (Acris Antibodies, Herford, Germany), an anti-plasminogen IgG developed in sheep (Acris Antibodies) at a 1:2000 dilution and a peroxidase-conjugated anti-sheep IgG (Sigma-Aldrich) at a 1:4000 dilution. These incubations were carried out in blocking solution at 37 °C for 1 h. Wells were washed three times with PBS between each step. Reactions were revealed with ortho-phenylene-diamine. A competition assay was performed in parallel including 25 mM of ε-aminocaproic acid (ε-ACA), a lysine analogue, during plasminogen incubation. Some wells were coated with BSA as a negative control. Optical densities (OD) were measured at 492 nm in a Microplate Absorbance Reader iMark (Bio-Rad, Hercules, USA). Each sample was analysed in triplicate.

In order to confirm plasminogen binding and characterize the parasitic protein bands responsible for this process, a western blot was employed [21]. To carry out the SDS-PAGE electrophoresis, 15 µg/well of AsL3C or AsL3ES were loaded into 12% polyacrylamide gels. Two µg of plasminogen were loaded as a positive control and the presence of the antigenic extracts was omitted in some wells to serve as a negative control. Once proteins were separated by molecular weight, some gels were developed with silver stain, while others were transferred to nitrocellulose membranes by semi-dry transfer to perform the western blot. Membranes were blocked with 2% BSA in wash buffer (PBS 0.05% Tween 20) at room temperature for 1 h and incubated with 10 µg/ml of human plasminogen (Acris Antibodies) in blocking solution at 4 °C overnight. Membranes were then successively incubated with an anti-plasminogen IgG developed in sheep (Acris Antibodies) at a 1:1000 dilution and a peroxidase-conjugated donkey anti-sheep IgG (Sigma-Aldrich) at a 1:2000 dilution in blocking solution at 37 °C for 1 h 30 min. Membranes were washed three times with wash buffer between each step. The reactions were revealed with 4-cloronaftol with peroxidase substrate. Gels and membranes were scanned in GS-800 Densitometer (Bio-Rad) and analysed with the Quantity One software v.4.6.5 (Bio-Rad). All assays were performed in triplicate.

### Immunofluorescence plasminogen binding assay

To locate plasminogen binding on the larval surface, an immunofluorescence assay was performed and analysed by confocal microscopy following the method described by Ramajo-Hernández et al. [21] with minor modifications. AsL3 previously fixed in 10% formaldehyde were successively incubated with 10 µg/ml of human plasminogen (Acris Antibodies) diluted in 1% BSA in PBS at room temperature for 2 h shaking and 10 µg/ml of a fluorescein isothiocyanate (FITC)-labelled anti-plasminogen antibody (Acris Antibodies) diluted in PBS at 4 °C shaking overnight in the dark. AsL3 were washed four times with PBS between each step. The presence of plasminogen was omitted in some assays as a negative control. In parallel, a competition assay with 40 mM of ε-ACA was carried out during plasminogen incubation. AsL3 were then observed under the 63× water immersion objective of a TCS SP2 DM IRB microscope (Leica, Wetzlar, Germany), using an Argon 488 laser and 500 nm filter at the microscopy facility of the Instituto de Recursos Naturales y Agrobiología de Salamanca (IRNASA-CSIC), Spain. All assays were performed in triplicate.

### Plasminogen activation assay

In order to evaluate the ability of both antigenic extracts to activate plasminogen and promote plasmin generation, a chromogenic assay was carried out according to the methodology described by González-Miguel et al. [19] and Fernandes et al. [22] with some modifications. One µg of AsL3C or AsL3ES was incubated in the presence of 1 µg of human plasminogen (Acris Antibodies), 3 µg of the plasmin chromogenic substrate S-2251 (Sigma-Aldrich) and a plasminogen activator [15 ng of tPA (Sigma-Aldrich) or 10 ng of uPA (Sigma-Aldrich)] in a final volume of 100 µl of PBS in multi-well microplates (Corning). In some wells the antigenic extract was replaced by BSA as negative control and in others the presence of plasminogen activators was omitted to study the ability of antigenic extracts to generate plasmin on their own. All the mixtures were maintained at 37 °C for 2 h 30 min and the hydrolysis of the substrate was analysed at 415 nm in a Microplate Absorbance Reader iMark (Bio-Rad). Each sample was analysed in triplicate.

### Two-dimensional (2-D) electrophoresis of AsL3C and AsL3ES extracts and immunoblot assays

For the characterisation of plasminogen-binding proteins from AsL3C and AsL3ES extracts, 2-D electrophoresis and plasminogen-ligand blotting were performed as previously described [19]. For that purpose, both antigenic extracts were purified using the ReadyPrep 2-D Cleanup Kit (Bio-Rad) and assayed in a 2-D electrophoresis. After re-suspending 60 µg of protein from each extract in 125 µl of a rehydration buffer [7 M urea, 2 M thiourea, 4% 3-((3-cholamidopropyl) dimethylammonio)-1-propanesulfonate (CHAPS)], the mixtures were incubated in the presence of ampholytes and dithiothreitol (DTT). Samples were applied to 7 cm IPG strips (Bio-Rad) with a linear pH range of 3–10 and once they were absorbed by the gel strip, proteins were separated in a first dimension depending on their isoelectric point (pI) using a Protean IEF Cell (Bio-Rad). After that, strips were reduced and alkylated using successively dilutions of DTT and iodoacetamide in equilibrated buffer [6 M urea, 2% SDS, 0.05 M Tris/HCl (1.5 M, pH 8.8), 30% glycerol]. The second-dimension separation by molecular weight (MW) was carried out in 12% polyacrylamide gels and proteins were detected by a silver stain. 2-D gels were scanned with the GS-800 Densitometer (Bio-Rad) and the resulting images were analysed with the Quantity One software v.4.6.5 (Bio-Rad).

To perform western blots, 2-D gels were transferred to nitrocellulose membranes using a semi-dry transfer in a Trans-Blot SD Semi-Dry Transfer cell (Bio-Rad). Membranes were then blocked with 2% BSA in wash buffer (PBS 0.05% Tween 20) for 1 h at room temperature and incubated with 10 µg/ml of human plasminogen (Acris Antibodies) in blocking solution at 4 °C overnight. Then, membranes were successively incubated with a sheep anti-human plasminogen IgG (Acris Antibodies) at a 1:1000 dilution and a peroxidase-conjugated donkey anti-sheep IgG (Sigma-Aldrich) at a 1:2000 dilution in blocking solution at 37 °C for 1 h 30 min. Between each step, membranes were washed three times with wash buffer. Plasminogen-binding spots were revealed with 4-chloronaphthol and matched to their corresponding 2-D gels using the PDQuest software v.8.0.1 (Bio-Rad). Plasminogen incubation was omitted in some membranes as negative controls. Membranes were scanned with the GS-800 Densitometer (Bio-Rad) and analysed with the Quantity One software v.4.6.5 (Bio-Rad). All assays were performed in triplicate.

### Mass spectrometry (MS), protein identification and bioinformatics analyses

Selected plasminogen-binding spots were manually excised from the silver stained 2-D gels and analysed by MS at the proteomics facility of the Servei Central de Suport a la Investigació Experimental (SCSIE) of the University of Valencia, Spain. Samples were digested with sequencing grade trypsin (Promega, Madison, USA) [23] and the resulting digestion mixture was dried in a vacuum centrifuge and resuspended in 7 µl of 2% acetonitrile (ACN), 0.1% trifluoroacetic acid (TFA), spotting 1 µl onto the target plate. After the droplets were air-dried at room temperature, 1 µl of matrix [10 mg/ml α-Cyano-4-hydroxycinnamic acid - α-Cyano-2,4-difluorocinnamic acid - α-Cyano-2,3,4,5,6-pentafluorocinnamic acid mixture (CHCA) (Sigma-Aldrich) in 0.1% TFA-ACN/H_2_O (1:1, v/v)] was added and allowed to air-dry at room temperature. The resulting mixtures were analysed in a 5800 Matrix-Assisted Laser Desorption/Ionization Time-Of-Flight (MALDI TOF/TOF) (AB Sciex, Framingham, USA) in positive reflectron mode. Five of the most intense precursors were selected for every position for the MS/MS analysis. MS/MS data were acquired using the default 1 kV MS/MS method with collision-induced dissociation (CID) active. The MS and MS/MS information was sent to MASCOT *via* the Protein Pilot (AB Sciex). The samples that did not have significant identification were analysed by liquid chromatography and tandem mass spectrometry (LC-MS/MS). Five µl of each sample were loaded onto a trap column (NanoLC Column, 3 µ C18-CL, 350 µm × 0.5 mm, Eksigent) and desalted with 0.1% TFA at 3 µl/min for 5 min. The peptides were then loaded onto an analytical column (LC Column, 3 µ C18-CL, 75 µm × 12 cm, Nikkyo) equilibrated in 5% ACN, 0.1% formic acid (FA). Elution was carried out with a linear gradient of 5 to 40% B in A (A: 0.1% FA; B: ACN, 0.1% FA) at a flow rate of 300 nl/min for 15 min. Peptides were analysed in a mass spectrometer nanoESIqQTOF (5600 TripleTOF, AB Sciex). Samples were ionized applying 2.8 kV to the spray emitter and analysed in a data-dependent mode. Survey MS1 scans were acquired from 350–1250 m/z for 250 ms. The quadrupole resolution was set to UNIT for MS2 experiments, which were acquired 100–1500 m/z for 50 ms in ‘high sensitivity’ mode. Up to 50 ions were selected for fragmentation after each survey scan. Dynamic exclusion was set to 15 s. The system sensitivity was controlled with 2 fmol of 6 proteins (LC Packings). Database searches were performed on the National Center for Biotechnology Information (NCBI) database with tryptic specificity allowing one missed cleavage and a tolerance on the mass measurement of 100 ppm in MS mode and 0.8 Da for MS/MS ions. Carbamidomethylation of Cys was used as a fixed modification and oxidation of Met and deamidation of Asn and Gln as variable modifications.

The molecular function and biological processes of the identified proteins were assigned according to the gene ontology database (http://www.geneontology.org) and the Swiss-Prot/UniProt database (http://beta.uniprot.org). Prediction of the secondary structures and three-dimensional modelling of the resulting sequences was conducted with the Swiss Model server [24] (http://swissmodel.expasy.org/). The 3-D models were visualized with the RasMol software v. 2.7.5.2.

### Statistical analysis

Plasminogen binding and plasminogen activation assays results were analysed with the Student’s t-test. Data are expressed as the mean ± standard deviation (SD) of three independent experiments. Significant differences were defined as a *P*-value of < 0.05 for a confidence level of 95%.

## Results

### AsL3C and AsL3ES bind plasminogen

**Fig. 1:**
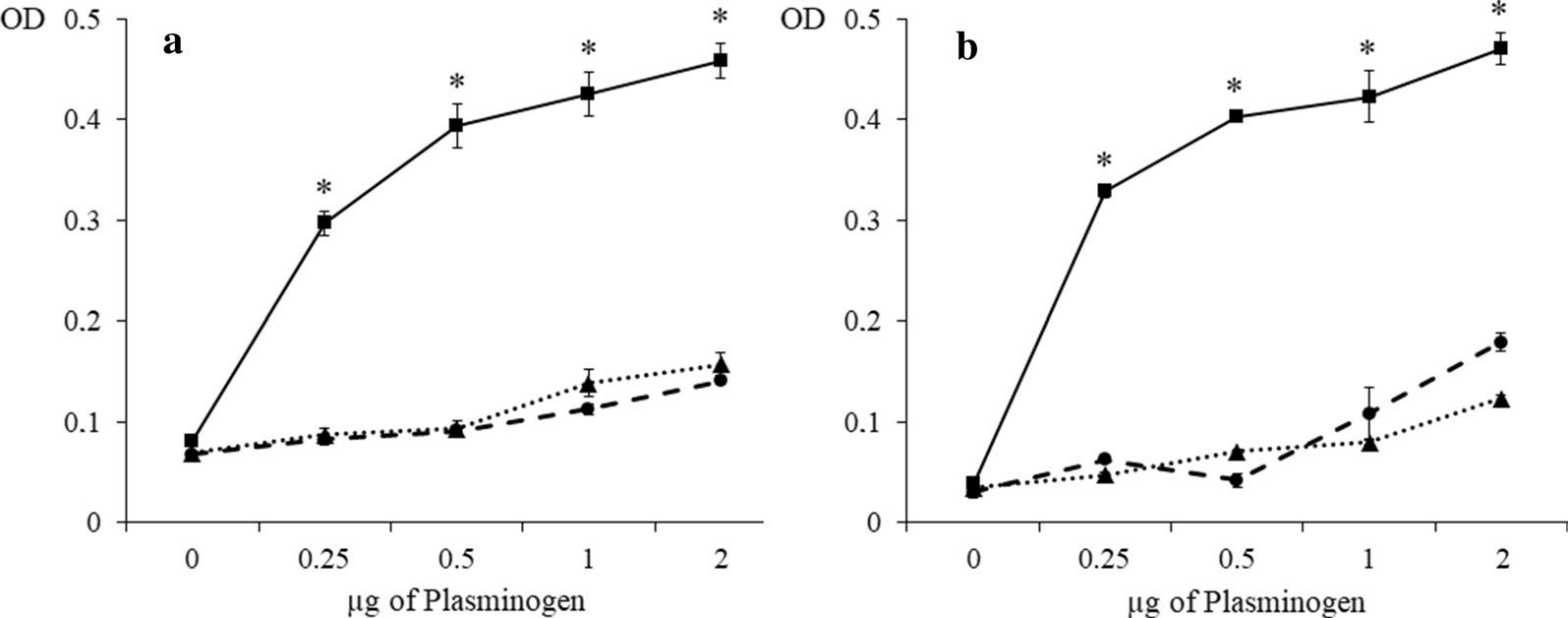
Plasminogen binding to AsL3C (**a**) and AsL3ES (**b**) by ELISA. Key: squares, 1 µg of AsL3C or AsL3ES incubated with increasing amounts of plasminogen (0–2 µg); circles, negative controls replacing the antigenic extracts by BSA; triangles, competition assays with 25 mM ε-ACA during plasminogen incubation. Each point represents the mean of three replicates ± SD. *P < 0.05

The ability of AsL3C and AsL3ES to bind plasminogen was assayed by ELISA. The results showed that both antigenic extracts were able to bind plasminogen since their optical densities were significantly higher than those obtained for the negative controls with BSA (AsL3C: *t*_(4)_ = 27.4955, *P* = 0.0001; *t*_(4)_ = 22.3828, *P* = 0.0001; *t*_(4)_ = 19.4370, *P* = 0.0001; *t*_(4)_ = 24.9594, *P* = 0.0001); AsL3ES: *t*_(4)_ = 75.8832, *P* = 0.0001; *t*_(4)_ = 193.3830, *P* = 0.0001; *t*_(4)_ = 23.1134, *P* = 0.0001; *t*_(4)_ = 37.6490, *P* = 0.0001). In addition, this binding was directly proportional to the amount of plasminogen (Fig. 1). The competition assay with ε-ACA showed similar values to the negative control both for AsL3C and AsL3ES, indicating the involvement of lysine residues in the plasminogen interaction (Fig. 1).

**Fig. 2:**
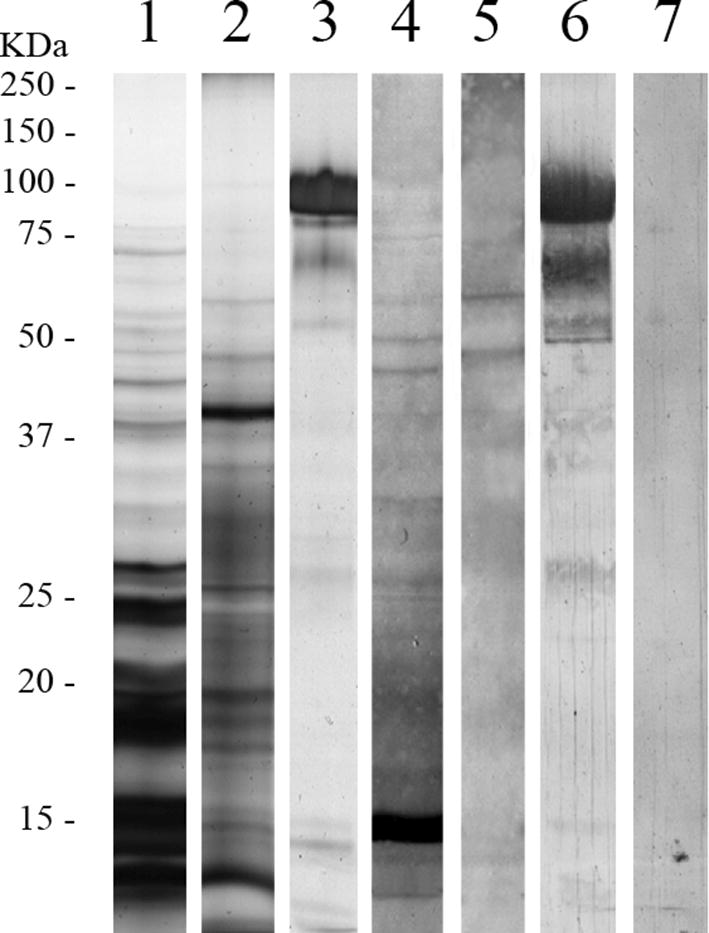
Plasminogen binding to AsL3C and AsL3ES by western blot. Lanes 1–3: SDS-PAGE with 15 µg of AsL3C (Lane 1), 15 µg of AsL3ES (Lane 2) and 2 µg of plasminogen (Lane 3); Lanes 4–7: western blot incubated with plasminogen: AsL3C (Lane 4), AsL3ES (Lane 5) plasminogen (Lane 6) and the negative control (Lane 7). The reference of molecular weights is indicated on the left

Plasminogen binding by AsL3C and AsL3ES was corroborated and characterized by western blot. Both antigenic extracts revealed plasminogen-binding bands that were absent in the negative control (Fig. 2). The SDS-PAGE showed approximately 30 bands for AsL3C and 20 for AsL3ES distributed in a broad MW range (between 10 and 100 kDa), of which 12 (10–75 kDa) and 5 (30–75 kDa) were revealed as plasminogen-binding bands in the immunoblots, respectively (Fig. 2).

### Plasminogen is attached to the larval surface

**Fig. 3:**
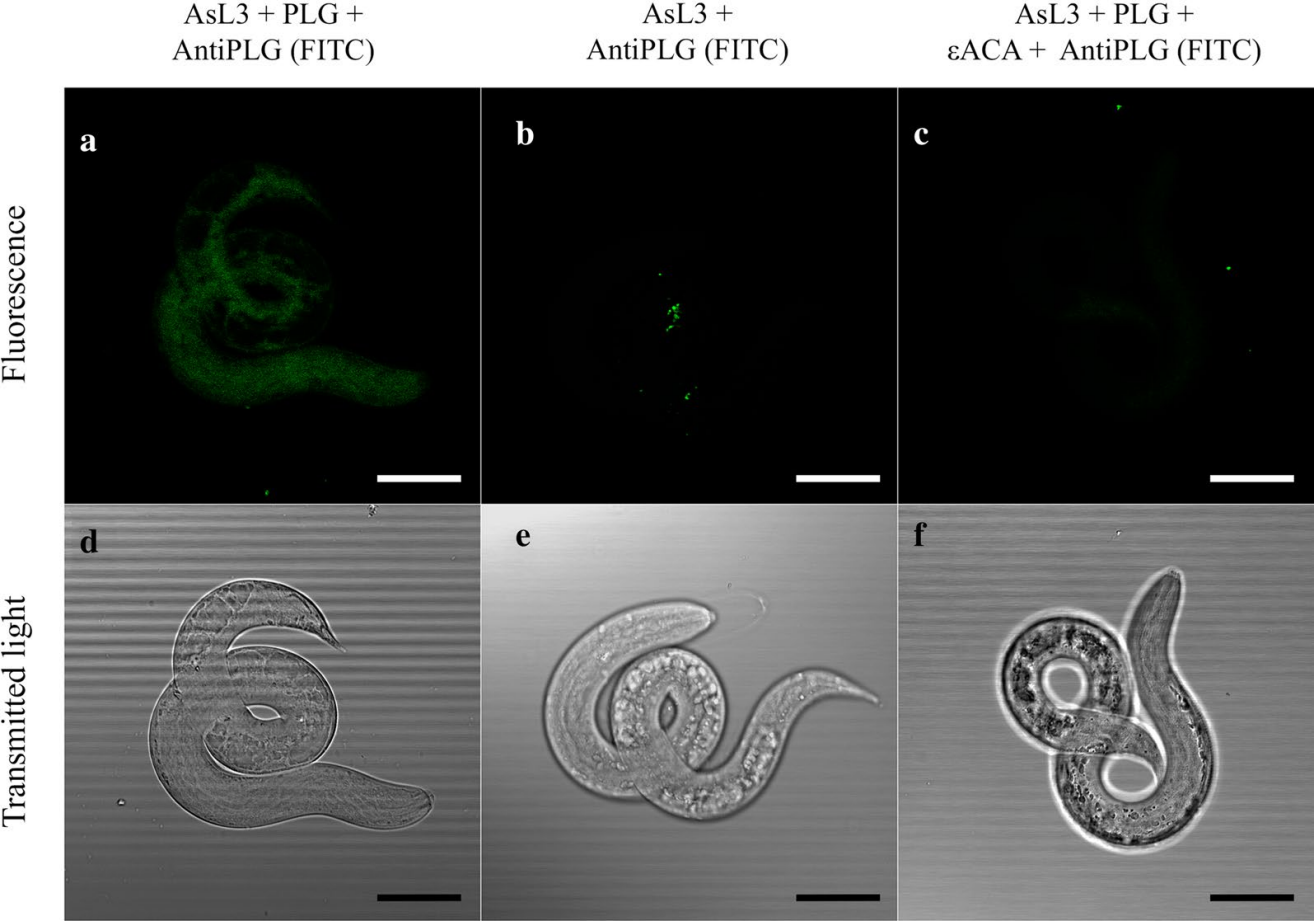
Plasminogen binding on the *A. suum* larval surface by immunofluorescence and confocal microscopy. **a**–**c** Fluorescence images from larvae surface. **a** Incubation with plasminogen and a (FITC)-labelled anti-plasminogen antibody. **b** The presence of plasminogen was omitted in the previous assay as a negative control. **c** Competition assay with 40 mM ε-ACA during plasminogen incubation. **d–f** Corresponding light microscopy images to **a**–**c**. *Scale-bars*: 25 µm

The location of plasminogen binding on the larval surface was analysed by immunofluorescence and confocal microscopy. Plasminogen binding was observed along the larval surface, mainly in the anterior part of the body. No binding was revealed in the negative control. The assay performed in the presence of ε-ACA showed a much lower binding level than that carried out in its absence, suggesting the participation of lysine residues in the plasminogen binding (Fig. 3).

### AsL3C and AsL3ES activate plasminogen and promote plasmin generation

**Fig. 4:**
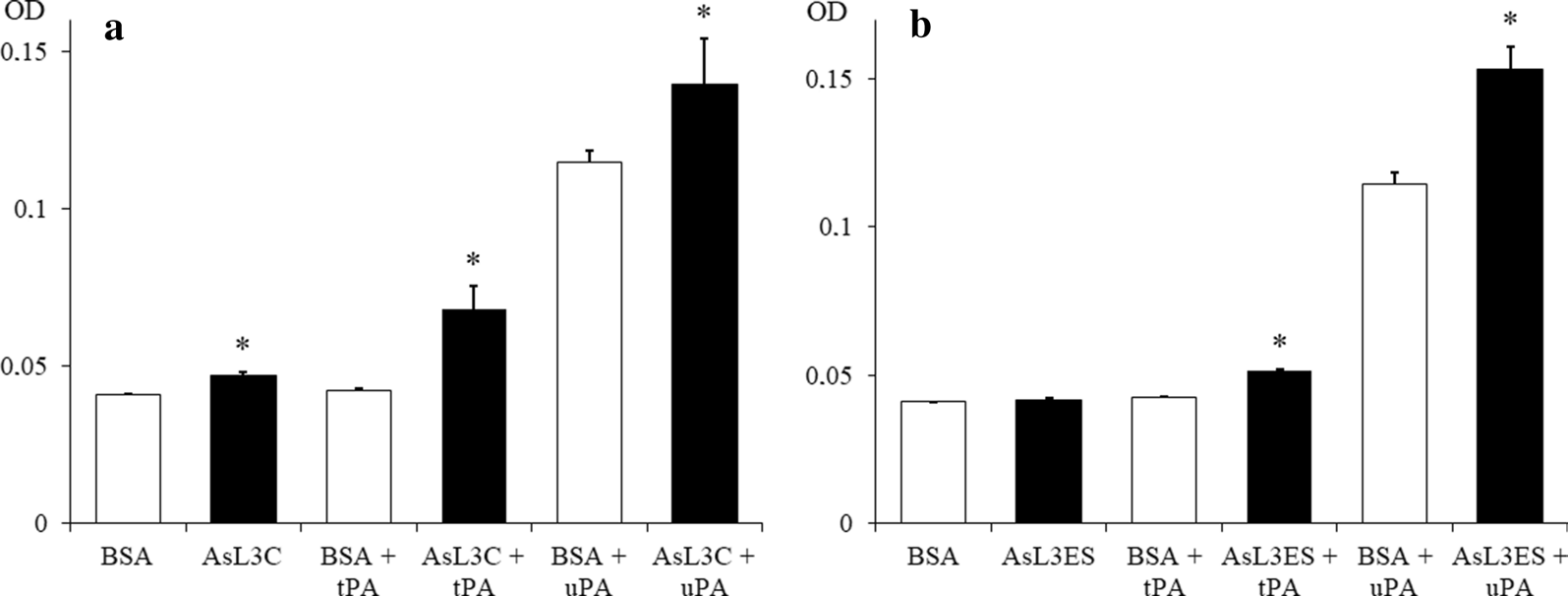
Plasminogen activation and plasmin generation by AsL3C (**a**) and AsL3ES (**b**). *Key*: filled bars, 1 µg of AsL3C or AsL3ES incubated with 1 µg of plasminogen, 3 µg of S-2251 and a plasminogen activator (15 ng of tPA or 10 ng of uPA); empty bars, negative controls replacing the antigenic extracts by BSA. Each point represents the mean of three replicates ± SD. The asterisk (*) indicates significant differences (*P* < 0.05) with the control groups on its left

The ability of AsL3C and AsL3ES to activate plasminogen and enhance plasmin generation was studied in a chromogenic assay. Both antigenic extracts activated plasminogen and promoted plasmin generation in the presence of both plasminogen activators, tPA and uPA, since the results obtained for these assays were significantly higher than those obtained by their corresponding negative controls [(AsL3C: (tPA) *t*_(4)_ = 5.8712, *P* = 0.0042; (uPA) *t*_(4)_ = 2.8378, *P* = 0.0470) (AsL3ES: (tPA) *t*_(4)_ = 19.0919, *P* = 0.0001; (uPA) *t*_(4)_ = 8.2024, *P* = 0.0012)] (Fig. 4). Comparing the results of both activators, the activation of plasminogen was higher by AsL3C and AsL3ES in the presence of tPA and uPA, respectively. Furthermore, AsL3C showed a slight ability to activate plasminogen in the absence of the activators since significant differences between the optical densities obtained by this experimental group and its negative control were found (*t*_(4)_ = 10.3923, *P* = 0.0005) (Fig. 4).

### Identification of plasminogen-binding proteins in AsL3C and AsL3ES

**Fig. 5:**
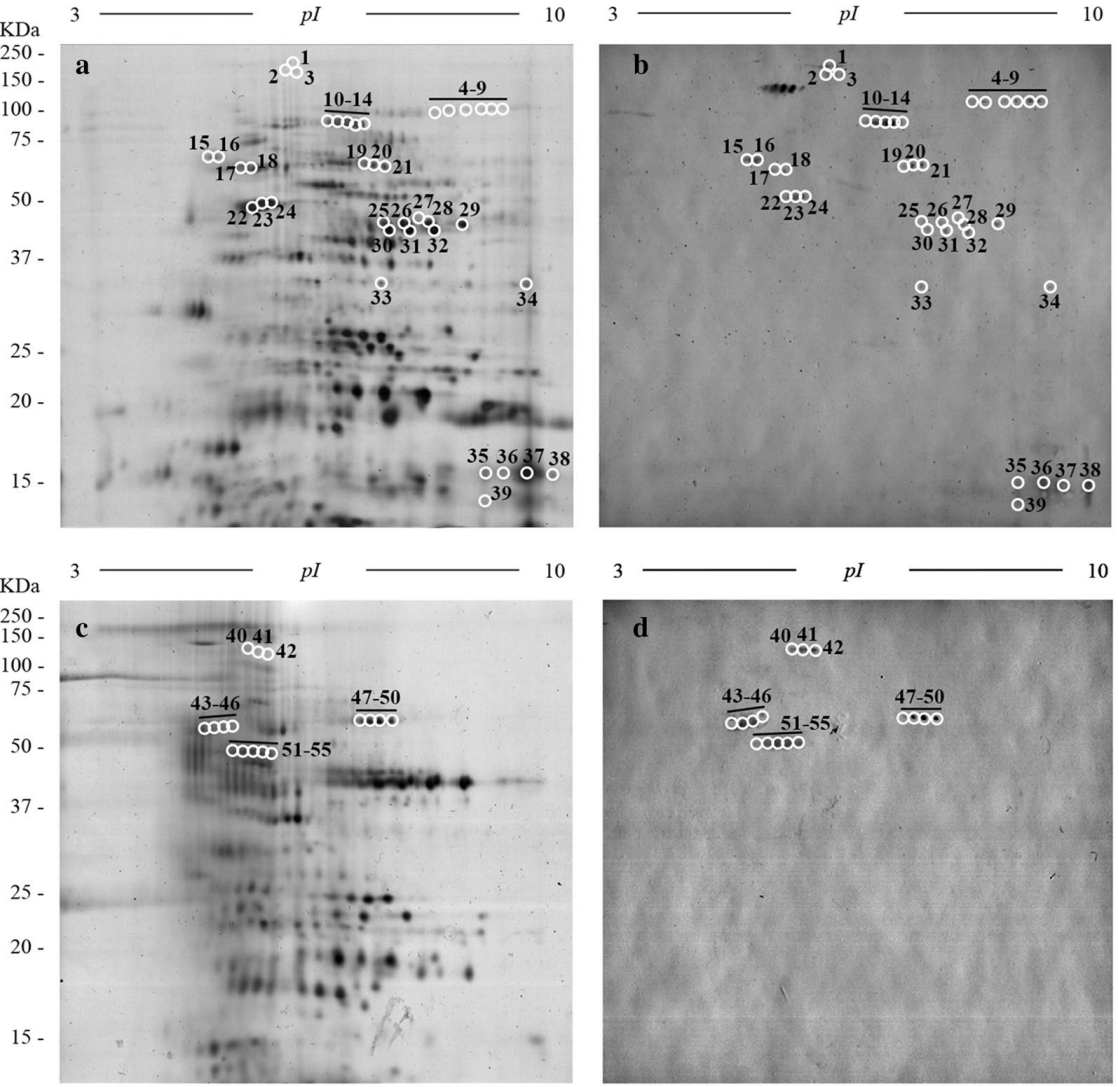
2-D images of AsL3C (**a**, **b**) and AsL3ES (**c**, **d**). **a**, **c** 2-D electrophoresis gels of 12% polyacrylamide and pH range of 3–10 revealed with silver stain. **b**, **d** Homologous western blots showing plasminogen-binding spots. The reference of molecular weights is indicated on the left. On the gel images, the reference of isoelectric point is indicated. Circled and numbered spots correspond to matched plasminogen-binding spots

In order to identify the proteins responsible for binding plasminogen, both antigenic extracts were analysed by 2-D electrophoresis and plasminogen-ligand blotting in a pH range of 3–10 and, subsequently, by MS. Silver stain of AsL3C 2-D gel revealed 560 spots with pIs between 3.3–9.7 and MWs between 12–176 kDa (Fig. 5a). The corresponding immunoblot revealed 54 plasminogen-binding spots with pIs between 5.1–9.5 and MWs between 13.7–176 kDa (Fig. 5b), representing a binding rate of 9.64% of total spots revealed. The AsL3ES 2-D gel showed a fewer number of spots (387) distributed in narrower ranges of pI (3.7–8.9) and MW (13.5–158 kDa) (Fig. 5c). In this case, only 16 spots were revealed as plasminogen-binding spots, with pIs between 5.1–7.1 and MWs between 48–109 kDa (Fig. 5d), representing a binding rate of 4.13%. In the case of the negative control, no spot was revealed in the membranes performed in the absence of plasminogen (not shown).

The matching of spots revealed by immunoblots with their homologous in the silver-stained 2-D gels allowed us to select a total of 41 and 16 plasminogen-binding spots in the AsL3C and AsL3ES proteomes, respectively. Among them, 30 spots (20 from AsL3C and 10 from AsL3ES) were manually excised from the 2-D gels and submitted to analysis by MS. All of them were identified, corresponding to 12 different proteins deposited in databases as *A. suum* proteins. The proteins identified were maguk p55 subfamily member 6, phosphoenolpyruvate carboxykinase, disulphide isomerase, ATP synthase subunit, glucose-6-phosphate isomerase, actin-2, fructose-bisphosphate aldolase 1, glyceraldehyde-3-phosphate dehydrogenase (GAPDH), 32 kDa beta-galactoside-binding lectin, 60s ribosomal protein l23, prion-like domain-bearing protein 25 and thachykinin-like peptides receptor 99d. Between 1 and 6 isoforms of each protein were identified. One of these proteins (glucose-6-phosphate isomerase) appeared in both antigenic extracts, 9 proteins derived from AsL3C and 2 proteins (prion-like domain-bearing protein 25 and thachykinin-like peptides receptor 99d) from AsL3ES. Table 1 shows the identity of these proteins with their corresponding NCBI accession code, their MWs and pIs, and the molecular function and biological process in which they are involved. Among these, 5 proteins were related to metabolic pathways, and 4 of them were linked with glycolytic processes.

**Table 1:** Plasminogen-binding proteins of AsL3C and AsL3ES identified by MS from analysed spots. All identifications belonged to *A. suum* proteins deposited in databases

Spot number	Antigenic extract	Accession code	Protein definition	Theoretical MW (kDa)	Theoretical pI	Molecular function	Biological process
1	AsL3C	ERG85667	Maguk p55 subfamily member 6	128.3	7.8	Binding activity	–
12, 13	AsL3C	Q05893	Phosphoenolpyruvate carboxykinase (GTP)	72.2	6.3	Phosphoenolpyruvate carboxykinase (GTP) activity	Gluconeogenesis
15, 16	AsL3C	CAK18211	Disulphide isomerase	55.6	4.9	Protein disulfide isomerase activity	Cell redox homeostasis
18	AsL3C	ERG82657	Atp synthase subunit	58.8	5.7	Proton transmembrane transporter activity	ATP synthesis coupled proton transport
20, 21, 49, 50	AsL3C, AsL3ES	ERG85026	Glucose-6-phosphate isomerase	80.1	9.1	Glucose-6-phosphate isomerase activity	Glycolytic process
23, 24, 36	AsL3C	ERG87158	Actin-2	41.8	5.3	Binding activity	Actin cytoskeleton organization
26, 28, 29	AsL3C	ERG79663	Fructose-bisphosphate aldolase 1	39.1	7.1	Fructose-bisphosphate aldolase activity	Glycolytic process
31, 32	AsL3C	ERG79426	Glyceraldehyde-3-phosphate dehydrogenase	38.5	7.7	Glyceraldehyde-3-phosphate Dehydrogenase activity	Glycolytic process
33, 34	AsL3C	ERG81473	32 kda beta-galactoside-binding lectin	31.8	6.4	Carbohydrate binding	–
35, 38	AsL3C	ERG81802	60s ribosomal protein l23	14.8	10.5	Structural constituent of ribosome	Translation
41, 42	AsL3ES	ERG86435	Prion-like domain-bearing protein 25	141.5	7.3	Catalytic activity	Carbohydrate metabolic process
44, 45, 51, 52, 54, 55	AsL3ES	ERG85099	Tachykinin-like peptides receptor 99d	87.8	4.9	G protein-coupled receptor activity	–

**Fig. 6:**
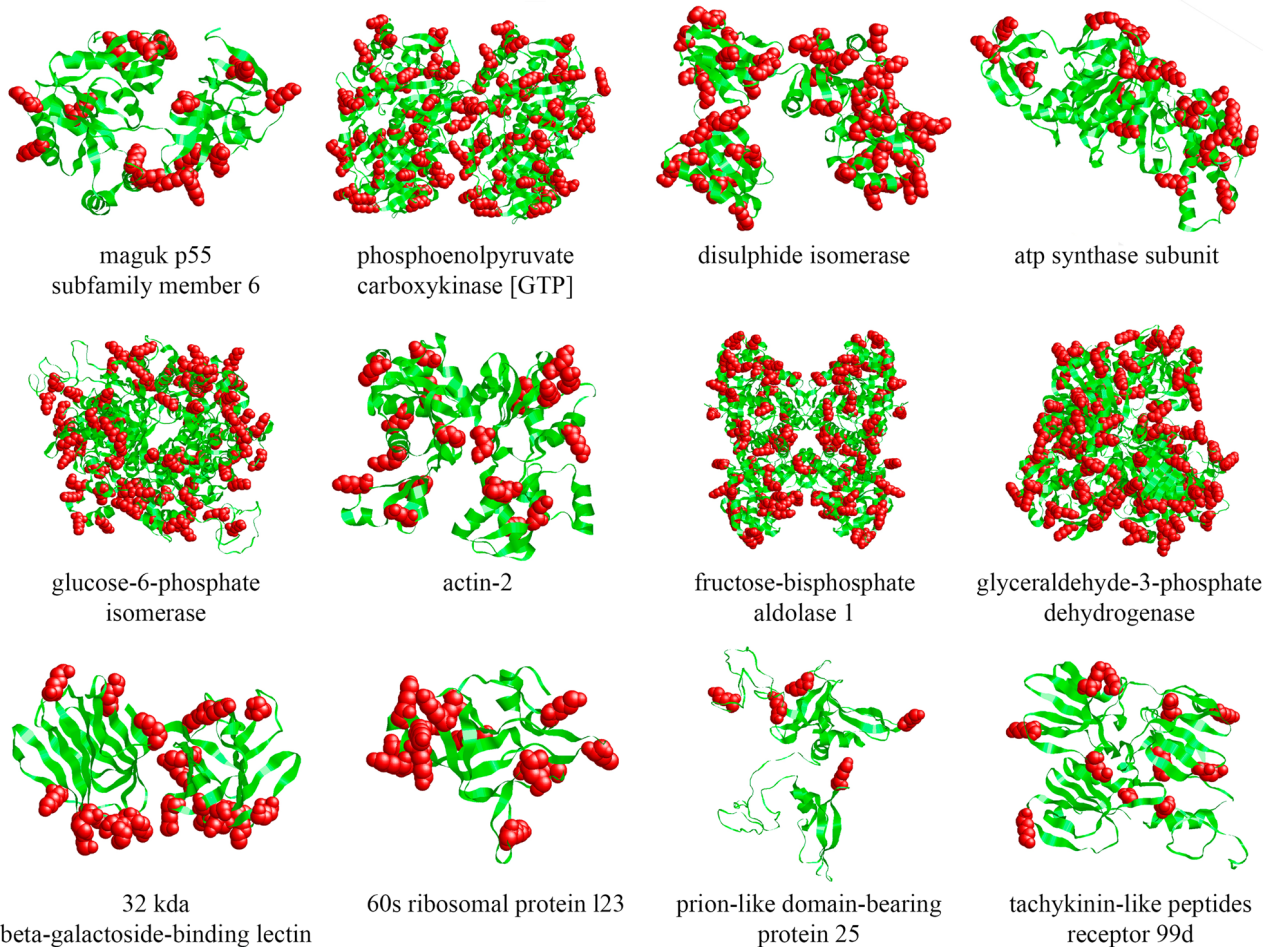
Molecular modelling of the plasminogen-binding proteins from AsL3 identified by MS. The secondary structure of the proteins was predicted with the Swiss-Model web server (http://swissmodel.expasy.org/) by analogy with the X-ray crystallography available models. The three-dimensional models of the molecules were visualized with the RasMol application v. 2.7.5.2. Lysine residues of proteins as possible plasminogen-binding sites are highlighted as red balls

Finally, *in silico* three-dimensional modelling of the identified plasminogen-binding proteins predicted their 3-D structures as it is shown in the Fig. 6. Lysine residues were highlighted and were visualized on the outside of the proteins as potential plasminogen-binding sites.

## Discussion

The plasmin broad substrate specificity makes this enzyme susceptible to be used by parasites to complement their own protease repertoire, which could facilitate their migration through the host tissues. In addition to parasites, other types of blood and/or tissue pathogens manipulate the host fibrinolytic system by means of the recruitment of plasminogen from the host plasma and its activation to plasmin [9, 10]. Despite *A. suum* being a parasite that spends most of its biological cycle in the intestinal tract of its host, its infective L3 stage carries out a wide migratory route through the host bloodstream [4]. For this reason, it was hypothesized that AsL3 interact with the host fibrinolytic system as a survival mechanism facilitating their intraorganic migration. In order to study this phenomenon, we decided to use cuticle and excretory/secretory antigenic extracts from the parasite larvae. Although the complete protein isolation of both extracts entails some limitations as a result of potential somatic products appearing in these compartments, we obtained two extracts enriched with cuticle and excretory/secretory proteins, respectively.

In the present study, we revealed the pro-fibrinolytic potential of the AsL3, due to the ability of their cuticle and excretory/secretory antigenic extracts to bind plasminogen in a lysine-dependent manner, which correlates with the results obtained for other helminth and protozoan parasites [10]. Plasminogen binding was located on the parasitic surface (Fig. 3), as previous studies have demonstrated for other parasites [21, 25]. This suggests that plasminogen-binding proteins are exposed to the larval surface, which could allow them to interact with plasminogen at the host-parasite interface. In addition, both antigenic extracts activated plasminogen and enhanced plasmin generation in the presence of host plasminogen activators (tPA and uPA), results that are consistent with those obtained for other parasites [21, 22, 26–31]. Plasmin generation by tPA was more effective in the presence of AsL3C, which could be due to the role of this molecule as the main activator responsible for the degradation of fibrin at a vascular level [11]. Hence AsL3 could use their surface associated plasminogen-binding proteins to promote plasmin generation in their immediate habitat while they are migrating through the bloodstream in order to avoid the clot formation. On the contrary, the generation of plasmin by uPA was higher in the presence of AsL3ES. uPA produces pericellular proteolysis since it binds to specific cellular receptors and activates cell-bound plasminogen [11]. Consequently, larvae could use their excretory/secretory plasminogen-binding proteins to enhance plasmin generation in specific cellular sites at a systemic level, as it has been postulated for other pathogens [30, 32]. Our results also showed that AsL3C induced plasmin generation by its own, in the absence of the plasminogen activators. Most of the studies carried out with parasitic extracts have revealed their dependence on the physiological plasminogen activators (tPA and/or uPA) in order to promote plasmin generation. This could indicate the presence of plasminogen activators in the AsL3 surface, as it has been already described for different genera of bacteria [14, 15], so that their identification could indicate a matter of future investigation. Regarding AsL3 pro-fibrinolytic potential, all these results suggest that there could be a combined action between both antigenic compartments at the host-parasite interface. Thus, AsL3 could use the proteolytic action of plasmin not only in their immediate environment, but also by directing it to those places where its activity is required at the pericellular level.

Plasmin produced by plasminogen activation can degrade fibrin networks and different proteins of the extracellular matrix [13], elements that could entail a barrier in the migration of parasites through the host tissues. The ability of AsL3 to enhance plasmin generation could be used by the parasite as a survival mechanism allowing it to degrade these components in order to facilitate its migration through the host tissues, as it has been postulated for other pathogens [27, 31, 33–36]. Regarding other survival-related functions, the degradation of proteins for nutrition, or of immunoglobulins and complement components for immune evasion mechanisms have also been suggested to be the result of the interaction between pathogens and the fibrinolytic system of their hosts [10]. Consequently, the plasmin generated by AsL3 could also contribute to the larval feeding or to modulate the host immune response. The latter could indicate that AsL3 can reach their definitive location at the small intestine of the host without being eliminated by the immune system during their migration through the bloodstream.

Apart from the effects related to the establishment and survival of the parasite in the host, the overproduction of plasmin by parasites has been linked with the appearance of pathological processes in the host, such as cell proliferation and migration, or inflammation [10, 37]. Intriguingly, one of the most distinctive lesions of ascariasis is the emergence of fibrotic, necrotic and hemorrhagic focus in the liver known as milk/white spots [38], which are accompanied by migratory trajectories of the parasitic larvae [6]. Even though it seems that these lesions are produced as a result of mechanical injury and inflammatory response induced by the larval migration in the liver [39], the overproduction of plasmin by the parasite could also contribute to this inflammatory phenomenon. These lesions not only cause damages on the health of the infected animals, but also comprise a significant problem for the porcine industry, since they produce less suitable livers for human consumption, producing economic losses for abattoirs [1]. Similar spots appear in the lungs, which are described as chronic reactions due to the larval migration [40], and in the intestinal mucosa, as a result of the larval penetration in the caecum and proximal colon [6].

In this study, 12 different proteins were identified as potential plasminogen receptors. Among them, GAPDH, fructose-bisphosphate aldolase 1 and actin-2 have been widely studied for their ability to interact with the fibrinolytic system of the host, as well as with the possible linkage of this interaction with the pathogen invasion processes [10]. Their roles as potential plasminogen receptors in a wide variety of unrelated biological groups show their high evolutionary conservation and the importance of plasminogen recruitment for pathogens in general and parasites in particular [10]. In addition, these proteins have been previously described as “moonlighting proteins” [41], which means that they are able to perform distinct functions depending on whether they are located inside or outside the cell, or in different compartments of the cell [42]. Following this idea, GAPDH and fructose-bisphosphate aldolase can function in the cytosol as glycolytic enzymes, while they could bind plasminogen outside the cell as part of the excretory/secretory system [43]. In this case, this secondary function would be possible due to the interaction between the so-called plasminogen kringle domains with the lysine residues of their receptors, which determines the phenomenon of plasminogen binding [44]. According to the 3-D models developed in this work, our results showed that lysine residues seem to be located externally in all the identified plasminogen-binding proteins, which would facilitate the accessibility of plasminogen (Fig. 6). Other proteins identified in this study that have been previously described as potential plasminogen receptors in other parasites are beta-galactoside-binding lectin and disulphide isomerase, which were found in the host-parasite interface of *Dirofilaria immitis* and *Fasciola hepatica*, respectively [29, 30]. The remaining proteins (maguk p55 subfamily member 6, phosphoenolpyruvate carboxykinase, ATP synthase subunit, glucose-6-phosphate isomerase, 60s ribosomal protein l23, prion-like domain-bearing protein 25 and thachykinin-like peptides receptor 99d) have been related to the plasminogen recruitment for the first time in this study. Interestingly, the ATP synthase subunit has a previous connection with plasminogen binding since different subunits of this protein bind angiostatin, a proteolytic fragment of plasminogen, in human cells [45].

## Conclusions

To our knowledge, we demonstrate for the first time the interaction between AsL3 and the fibrinolytic system of the host. Both cuticle and excretory/secretory antigenic extracts of the parasite bind plasminogen and enhance plasmin generation, which could facilitate the intraorganic migration of the parasite. As previously mentioned, *A. suum* is currently considered as a model to study *A. lumbricoides*. This is possible because both species have the same life-cycle and there are cross infections and gene flow between them, since they are able to produce hybrids [46–48]. This fact allows to extrapolate our results to the knowledge of the molecular mechanisms governing a human health problem of increasing importance that affects about a billion people in the world, such as human ascariasis. Future studies aimed at unravelling the molecular relationships between *Ascaris* roundworms and their hosts in an early phase of infection could be of paramount importance in order to design new tools that allow us to control these parasitosis before the adult worms are established in their definitive location.

## Data Availability

Data supporting the conclusions of this article are included within the article. The datasets used and/or analysed during the present study are available from the corresponding author upon reasonable request.

## Abbreviations

L3: third-stage larvae
tPA: tissue plasminogen activator
uPA: urokinase-type plasminogen activator
AsL3C: cuticle antigenic extract of the L3 of *A. suum*
AsL3ES: excretory/secretory antigenic extract of the L3 of *A. suum*
AsL3: third-stage larvae of *A. suum*
PBS: phosphate-buffered saline
CTAB: cetyl trimethyl ammonium bromide
ELISA: enzyme-linked immunosorbent assay
BSA: bovine serum albumin
ε-ACA: ε-aminocaproic acid
OD: optical densities
FITC: fluorescin isothiocyanate
2-D: two-dimensional
CHAPS: 3-((3-cholamidopropyl) dimethylammonio)-1-propanesulfonate
DTT: dithiothreitol
pI: isoelectric pint
MW: molecular weight
MS: mass spectrometry
ACN: acetonitrile
TFA: trifluoroacetic acid
CHCA: α-Cyano-4-hydroxycinnamic acid - α-Cyano-2,4-difluorocinnamic acid - α-Cyano-2,3,4,5,6-pentafluorocinnamic acid mixture
MALDI TOF/TOF: Matrix-Assisted Laser Desorption/Ionization Time-Of-Flight
CID: collision-induced dissociation
LC-MS/MS: liquid chromatography and tandem mass spectrometry
FA: formic acid
NCBI: National Center for Biotechnology Information
SD: standard deviation
GAPDH: glyceraldehyde-3-phosphate dehydrogenase
